# Diagnosis and treatment outcomes from prebronchodilator spirometry performed alongside lung cancer screening in a Lung Health Check programme

**DOI:** 10.1136/thorax-2022-219683

**Published:** 2023-03-27

**Authors:** Claire Bradley, Alison Boland, Louisa Clarke, Naomi Dallinson, Claire Eckert, Deborah Ellames, Jonathan Finn, Rhian Gabe, Neil Hancock, Martyn PT Kennedy, Jason Lindop, Ayad Mohamed, Gabriel Mullen, Rachael L Murray, Suzanne Rogerson, Bethany Shinkins, Irene Simmonds, Sara Upperton, Anne Wilkinson, Philip A Crosbie, Matthew EJ Callister

**Affiliations:** 1 Department of Respiratory Medicine, Belfast Health and Social Care Trust, Belfast, UK; 2 Department of Respiratory Medicine, Leeds Teaching Hospitals NHS Trust, Leeds, UK; 3 Community Respiratory Team, Leeds Community Healthcare NHS Trust, Leeds, UK; 4 Leeds Diagnosis and Screening Unit, Institute of Health Sciences, University of Leeds, Leeds, UK; 5 Barts Clinical Trials Unit, Centre for Evaluation and Methods, Wolfson Institute of Population Health, Queen Mary University of London, London, UK; 6 Department of Research and Innovation, Leeds Teaching Hospitals NHS Trust, Leeds, UK; 7 Oxford Centre for Respiratory Medicine, Oxford University Hospitals NHS Foundation Trust, Oxford, UK; 8 Lifespan and Population Health, School of Medicine, University of Nottingham, Nottingham, UK; 9 Division of Infection, Immunity and Respiratory Medicine, University of Manchester, Manchester, UK

**Keywords:** COPD epidemiology

## Abstract

**Introduction:**

Incorporating spirometry into low-dose CT (LDCT) screening for lung cancer may help identify people with undiagnosed chronic obstructive pulmonary disease (COPD), although the downstream impacts are not well described.

**Methods:**

Participants attending a Lung Health Check (LHC) as part of the Yorkshire Lung Screening Trial were offered spirometry alongside LDCT screening. Results were communicated to the general practitioner (GP), and those with unexplained symptomatic airflow obstruction (AO) fulfilling agreed criteria were referred to the Leeds Community Respiratory Team (CRT) for assessment and treatment. Primary care records were reviewed to determine changes to diagnostic coding and pharmacotherapy.

**Results:**

Of 2391 LHC participants undergoing prebronchodilator spirometry, 201 (8.4%) fulfilled the CRT referral criteria of which 151 were invited for further assessment. Ninety seven participants were subsequently reviewed by the CRT, 46 declined assessment and 8 had already been seen by their GP at the time of CRT contact. Overall 70 participants had postbronchodilator spirometry checked, of whom 20 (29%) did not have AO. Considering the whole cohort referred to the CRT (but excluding those without AO postbronchodilation), 59 had a new GP COPD code, 56 commenced new pharmacotherapy and 5 were underwent pulmonary rehabilitation (comprising 2.5%, 2.3% and 0.2% of the 2391 participants undergoing LHC spirometry).

**Conclusions:**

Delivering spirometry alongside lung cancer screening may facilitate earlier diagnosis of COPD. However, this study highlights the importance of confirming AO by postbronchodilator spirometry prior to diagnosing and treating patients with COPD and illustrates some downstream challenges in acting on spirometry collected during an LHC.

**WHAT IS ALREADY KNOWN ON THIS TOPIC**

- Between 10% and 20% of people attending for low-dose CT screening for lung cancer have unexplained symptomatic airflow obstruction and, thus, may have undiagnosed chronic obstructive pulmonary disease (COPD), but few studies have looked at what happens to people following this initial prebronchodilator spirometry.

**WHAT THIS STUDY ADDS**

- This study describes downstream events for people found to have unexplained airflow obstruction at a Lung Health Check who were referred to a Community Respiratory Team for further assessment and treatment. About one-third of referred people declined assessment, and of those seen and undergoing postbronchodilator spirometry, 29% did not have airflow obstruction. Of all participants undergoing Lung Health Check spirometry, 2.3% commenced appropriate new pharmacotherapy as a result and 0.2% entered pulmonary rehabilitation.

**HOW THIS STUDY MIGHT AFFECT RESEARCH, PRACTICE OR POLICY**

- Measuring spirometry alongside lung cancer screening offers the possibility of diagnosing COPD earlier. However, this study demonstrates the importance of checking postbronchodilator spirometry in this population and illustrates some of the ‘real-world’ challenges in actioning these findings. Further research is needed to optimise investigation and management of this population and to measure eventual clinical outcomes.

## Introduction

Chronic obstructive pulmonary disease (COPD) is a worldwide health problem that causes significant morbidity and mortality^1 2^ and shares the common risk factor of smoking with lung cancer. It is widely recognised that COPD is underdiagnosed,^3 4^ but screening for the disease in asymptomatic adults is not recommended by either the United Kingdom National Screening Committee^5^ or the United States Preventive Service Task Force (USPSTF).^6^ In comparison, the most recent Global Obstructive Lung Disease (GOLD) guidelines advocate active case finding in individuals with symptoms and/or risk factors, alongside aggressive identification and management of coexisting comorbidities.^7^

In the United Kingdom, several programmes have offered low-dose CT (LDCT) screening for lung cancer as part of a Lung Health Check (LHC), whereby a number of interventions (screening, prebronchodilator spirometry, smoking cessation) are offered as a bundle to improve lung health. Studies have reported between 10% and 20% of screening attendees having symptomatic undiagnosed airflow obstruction (AO) picked up by these programmes.^8–10^ However, there is limited evidence of subsequent downstream events, such as the proportion of individuals who undergo postbronchodilator spirometry as a formal diagnostic test for COPD. Only one study has reported treatment outcomes in this context, with 11% of individuals commencing new pharmacotherapy and 2% entering a pulmonary rehabilitation programme.^11^ Here, we present the downstream clinical assessment and management as a result of spirometry offered as part of an LHC in the Yorkshire Lung Screening Trial (YLST).

## Methods

### YLST study design

The protocol of the YLST has previously been published.^12^ Briefly, people aged 55–80 in Leeds were invited to telephone-based risk assessment for lung cancer and, if eligible for screening, were invited for a face-to-face LHC. These were provided in mobile units in convenient community locations and comprised LDCT screening, prebronchodilator spirometry and, where appropriate, an immediate opt-out consultation with a colocated smoking cessation practitioner (including Nicotine Replacement Therapy/pharmacotherapy, ongoing behavioural support and 4-week carbon monoxide validation for quitters). YLST was approved by the Health Research Authority following review by Research Ethics Committee (reference 18/NW/0012) and is registered with the ISRCTN (reference ISRCTN42704678).

### Data collection during the LHC

General practitioner (GP)-entered codes for a previous diagnosis of COPD were extracted from primary care electronic healthcare records; all other parameters were self-reported. Previous respiratory diagnoses, COPD assessment test scores (CAT), modified Medical Research Council dyspnoea score, WHO performance status and the presence of COPD-defining symptoms (exertional breathlessness, chronic cough, regular sputum production, wheeze, frequent winter bronchitis)^13^ were recorded for each participant. Measurements of prebronchodilator forced expiratory volume in 1 s (FEV_1_) and forced vital capacity (FVC) were performed with AO defined as an FEV_1_/FVC ratio of less than 0.7, and restrictive spirometry defined as an FEV_1_/FVC ratio of ≥0.7 and an FVC of <80% predicted.

### Communication of results and referral to the Community Respiratory Team

Criteria for referral of participants with possible undiagnosed COPD to the Leeds Community Respiratory Team (CRT) were: no COPD code on primary care record; no self-reported asthma; AO on spirometry (FEV_1_/FVC <0.7) and any self-reported COPD-defining symptom. For the first 2 months of the programme (November and December 2018), participants were referred irrespective of their FEV_1_ per cent-predicted value. Following review of referral numbers, from January 2019 onwards, an additional criterion of FEV_1_ less than 80% predicted was added. Referrals to the CRT were made by secure email communication after each round of LHCs (approximately monthly). Spirometry results including reference to the presence or absence of prebronchodilator AO and referral to the CRT where appropriate were communicated to the participant’s GP by electronic letter. A separate letter was sent to appropriate participants explaining the rationale for referral to the CRT within 4 weeks of their LHC.

### Community Respiratory Team review

Most referred participants were initially contacted by telephone by the CRT to invite them for a respiratory assessment. Those not contactable by phone were sent a letter asking them to contact the CRT to arrange an appointment; if there was no response within a month, they were discharged back to their GP. A questionnaire template was setup within the CRT electronic patient record (SystmOne, TPP) to record clinical information and outcomes from the assessment. Postbronchodilator spirometry was recorded for a proportion of participants referred to the service.

### COVID-19

The baseline round of YLST ran from November 2018 until February 2021. YLST was paused between March and June 2020 due to the COVID-19 pandemic, and when the programme recommenced in July 2020, spirometry was omitted from ongoing LHCs. Similarly, the CRT assessment of participants referred from screening was paused in March 2020 and did not restart again after the pandemic, with all those who remained on the waiting list redirected to their GP. Due to the lag between LHC visit and CRT assessment, referrals from YLST to the CRT were significantly affected for all screening rounds from October 2019 onwards. This analysis is, therefore, limited to participants who were screened between November 2018 and September 2019.

### Review of outcomes and analysis

Primary care records for people resident in Leeds are visible to secondary care users through the shared Leeds Care Record, except in instances where the patient has specifically asked for their record not to be available or where the patient is deceased. The primary care records of all participants referred to the CRT were reviewed from March to May 2021. The presence of a primary care code for COPD was determined, together with prescriptions for inhaled medications. Inhaled medications issued prior to the LHC visit were recorded, including whether the prescription was active at the time of the LHC visit (ie, recently issued) or had been previously discontinued. Similarly inhaled medication initiated since the LHC visit was recorded, including whether the prescription was active or not (defined as medication issued since January 2021). Appropriate statistical tests were used (Mann-Whitney U test and Fisher’s exact test) based on skewed data and categorical labels, and all statistical analyses were performed using GraphPad Prism V.5.00.

## Results

Between November 2018 and March 2020, 4510 participants attended for an LHC and underwent LDCT screening, of whom 3920 (87%) had prebronchodilator spirometry. Considering only those attending an LHC between November 2018 and September 2019 (ie, prior to the impact of COVID-19 on the referral pathway), 2786 participants underwent LDCT screening of whom 2391 (86%) had prebronchodilator spirometry measured on the mobile units. Of these, 505 had a COPD code recorded in their primary care record, and 145 self-reported a history of asthma.

**Figure 1:**
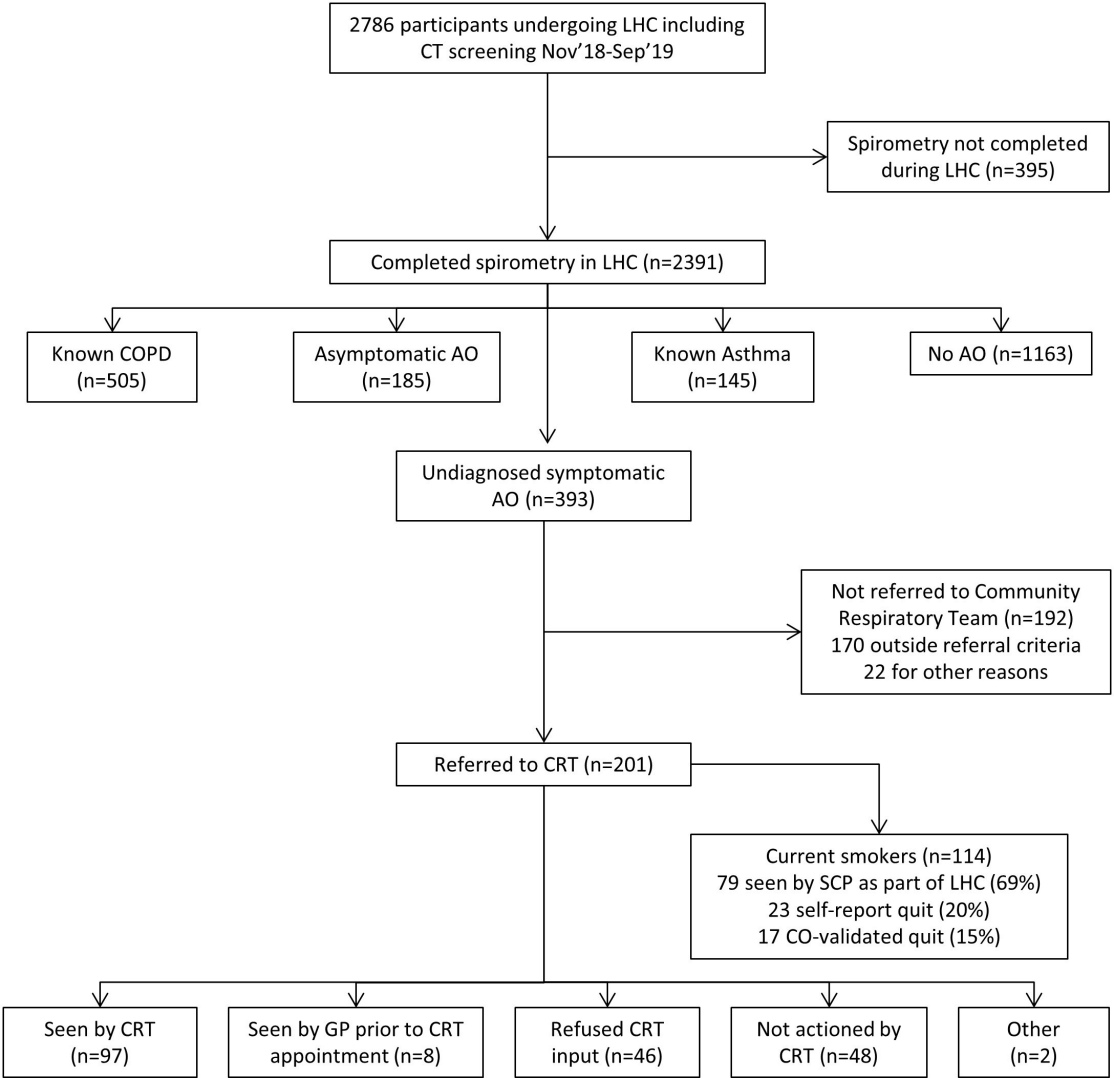
Consort diagram for participants attending a Lung Health Check between November 2018 and September 2019. AO, airflow obstruction, CRT, community respiratory team; LHC, lung health heck; SCP, smoking cessation practitioner.

Of the 1741 participants without a prior diagnosis of COPD or asthma, 1163 (67%) had no AO (1086 normal spirometry, 77 restrictive spirometry, mean [± SD] CAT score was 8.1±5.9 for this group as a whole) and 578 (33%) were found to have AO (185 asymptomatic, 393 symptomatic). The mean (±SD) CAT score was 4.5±3.3 for the asymptomatic AO group and 10.4±5.8 for the symptomatic AO group. Of the 393 with undiagnosed symptomatic AO, 192 were not referred to the CRT (170 because they did not fulfil the additional criteria of FEV1_1_<80% predicted introduced from January 2019 onwards, and 22 for a variety of other reasons - the the most common being referral to the lung cancer pathway). The remaining 201 participants were referred to the CRT; a consort diagram is shown in figure 1.

At the time of their LHC, all participants were asked whether they had a previous history of COPD, emphysema or bronchitis. Overall, 43 of the 201 people who fulfilled the referral criteria (21%) reported one or more of these conditions (bronchitis 34, COPD 9, emphysema 1), and 114 of the 201 (57%) were current smokers. All had been offered an immediate consultation with an on-site smoking cessation practitioner (SCP) at the time of their initial LHC. Seventy nine people were seen by the SCP at the time of their visit (69% of those offered) of whom 23 later reported a successful quit with 17 validated by carbon monoxide measurement (20% and 15% of eligible current smokers respectively).

Ninety seven participants were eventually seen by the CRT (48% of all those referred). Eight people were contacted by the CRT to arrange an appointment, but had already seen their GP since the LHC and had undergone assessment and management, so were not offered an appointment. Forty six people declined assessment, either refusing during the initial telephone call or failing to respond to written invitations. Fifty people were not contacted by the CRT or not seen for other reasons (44 due to logistical failure in the referral process, 4 were discharged having not been invited by the time the service closed due to COVID-19, 1 reported a diagnosis of asthma during his initial CRT telephone call, 1 was under investigation for lung cancer and was not contacted). Demographic and clinical characteristics of the cohort according to invitation/response are shown in table 1. There were no differences in demographic or clinical parameters between those who declined review and those seen (either by the CRT or their GP in advance of CRT contact).

**Table 1:** Demographics and clinical information regarding participants eligible for Community Respiratory Team referral for possible new COPD

	Seen by Community Respiratory Team (or GP)		Declined referral/failed to respond		Not invited/not seen for other reasons		Comparison seen vs decline/refused (p value)
Number of participants, % of overall eligible cohort	105	52%	46	23%	50	25%	
Age, mean, SD	68.7	7.2	66.4	6.9	66.2	6.6	0.074
Male, n, %	56	53%	28	61%	29	58%	0.477
**Ethnicity, n, %**							ND
White	103	98%	44	96%	46	92%	
Non-white (Black/Asian/Other)	2	2%	1	2%	4	8%	
Prefer not to say/no data	0	0%	1	2%	0	0%	
IMD decile, IQR (lower=more deprived)	4	1–7	2	1–5.25	3	1–5.25	0.238
IMD rank, median, IQR (lower=more deprived)	9864	1802–20371	4189	2104–16478	6898	1650–16818	0.443
**Education, n, %**							0.335
No qualifications, left school at or before 15	67	64%	27	59%	34	68%	
O levels, CSEs or equivalent	19	18%	13	28%	7	14%	
A-levels or above	19	18%	6	13%	9	18%	
**mMRC dyspnoea score, n, %**							1.000
0–1	85	81%	37	80%	44	88%	
2–4	20	19%	9	20%	6	12%	
**WHO performance status**							0.234
0–1	97	92%	39	85%	46	92%	
2–3	8	8%	7	15%	4	8%	
**COPD CAT score, median, IQR**	11	8–15	10	7–16.25	11	7–14	0.892
**Smoking status**							0.859
Current (within the last month or CO≥6 ppm), n, %	60	57%	25	54%	29	58%	
Ex-smoker, n, %	45	43%	21	46%	21	42%	
**Spirometry, mean, SD**							
FEV1 (L)	1.7	0.5	1.8	0.6	1.7	0.5	0.620
FEV1 %	69	17	67	17	66	10	0.780
FEV1/FVC ratio	0.6	0.07	0.6	0.07	0.61	0.07	0.648
**Emphysema on CT, n, %**							0.556
None/trivial	53	50%	19	41%	22	44%	
Mild/moderate/severe/very severe	45	43%	24	52%	25	50%	
Unclear/not reported	7	7%	3	7%	3	6%	

Statistical tests refer to comparison between those seen by the Community Respiratory Team or by their GP prior to CRT contact, vs those declining referral or failing to respond.

CAT, COPD assessment test; COPD, chronic obstructive pulmonary disease; FEV_1_, forced expiratory volume in 1 second; FVC, forced vital capacity; IMD, index of multiple deprivation; IQR, interquartile range; mMRC, Modified Medical Research Council.

**Figure 2:**
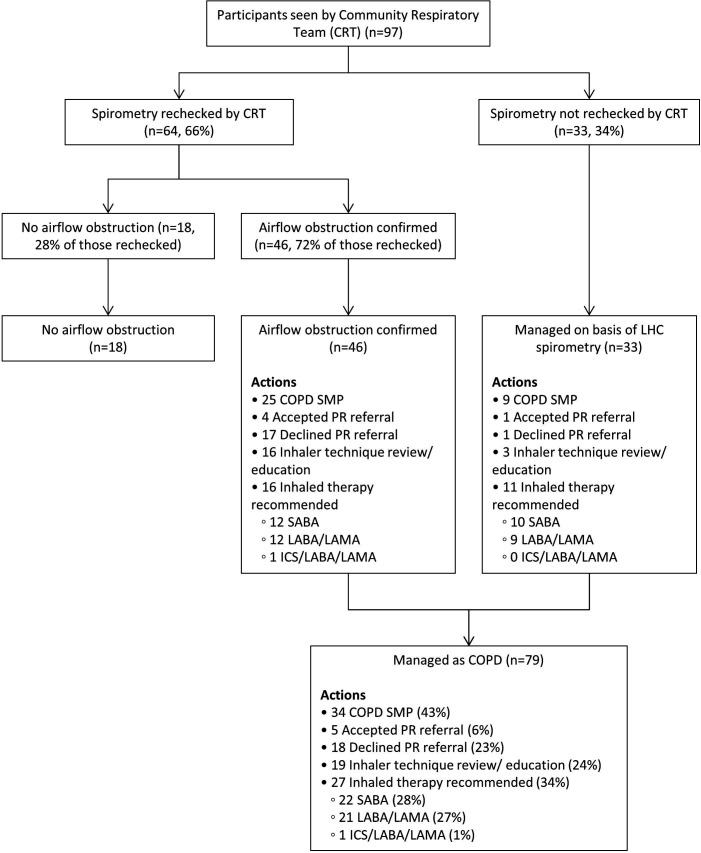
Outcomes for participants seen by the Community Respiratory Team. CRT, community respiratory team; ICS, inhaled corticosteroid; LABA, long acting beta agonist; LAMA, long acting muscarinic antagonist; LHC, lung ealth heck; PR, pulmonary rehabilitation; SABA, short acting beta agonist; SMP, self-management plan.

Outcomes for those seen and assessed by the CRT are shown in figure 2. Two thirds of those seen had post-bronchodilator spirometry checked with AO confirmed in 72%, but not shown in 28% of participants thereby excluding COPD. The 33 participants seen by the CRT who did not have repeat spirometry were managed on the basis of values measured during their LHC. Of the eight participants seen and assessed by their GP prior to CRT contact, 4 had post-bronchodilator spirometry by the GP confirming AO, 2 had normal post-bronchodilator spirometry and one had no record of spirometry in primary care record. One participant had died and thus their primary care record was not available for review. Thus of all 70 participants who had post-bronchodilator spirometry checked after the initial LHC for whom results are available, fifty (71%) had confirmed AO and 20 (29%) did not.

Outcomes for the 97 participants seen by the CRT are shown in figure 2. Forty six people had post-bronchodilator spirometry confirming AO, and 33 had no spirometry but were managed based on pre-bronchodilator spirometry measured at the LHC. Considering these two groups together, 34 people (43%) had record of a COPD self-management plan, and inhaled medication was recommended for 27 (34%), 22 (28%) short-acting beta agonist (SABA), 21 (27%) combination long acting beta-agonist/long acting antimuscarinic antagonist (LABA/LAMA). Nineteen participants underwent inhaler technique review or education, and 23 were offered pulmonary rehabilitation, but only 5 (22% of those offered) took up this referral.

The results of primary care record review for all 201 participants referred to the CRT are shown in table 2; the record was not accessible for 13 participants either because they had died, or because they had requested that this information not be available for secondary care review. Excluding those in whom subsequent post-bronchodilator spirometry ruled out COPD, 59 participants had a COPD code recorded in their primary care record in early 2021 (35% of all those with access to primary care records). The proportion of people with a COPD code was higher in those seen by the CRT (58%) than in those who refused assessment (17%) or those who were not contacted by the service (12%). Interestingly, reviewing those 20 participants where post-bronchodilator spirometry excluded COPD, 7 (35%) still had a GP COPD code present in their primary care record.

**Table 2:** Primary care COPD code and inhaled medication pre- and post-Lung Health Check visit

	Seen by CRT (or GP) excluding normal spiro		CRT/GP spirometry showed no obstruction		Declined referral/failed to respond		Not invited/not seen for other reasons		All excluding CRT/GP normal spiro	
Number of participants, % of overall eligible cohort	85	42%	20	10%	46	23%	50	25%	181	90%
Number without access to primary care record	5		0		4		4		13	
Number with access to primary care record	80		20		42		46		168	
GP COPD code, n, % of those with visible record	46	58%	7	35%	7	17%	6	13%	59	35%
**Inhalers prior to LHC** (% those with visible record)										
SABA (ceased in parentheses), n, %	16 (4)	20% (5%)	4 (2)	20% (10%)	5 (9)	12% (21%)	6 (5)	13% (11%)	27 (18)	16% (11%)
LAMA/LABA (ceased in parentheses), n, %	0 (0)	0% (0%)	0 (0)	0% (0%)	0 (0)	0% (0%)	1 (0)	2% (0%)	1 (0)	1% (0%)
ICS/LAMA/LABA (ceased in parentheses), n, %	0 (0)	0% (0%)	0 (0)	0% (0%)	0 (0)	0% (0%)	0 (0)	0% (0%)	0 (0)	0% (0%)
ICS/LABA (ceased in parentheses), n, %	1 (1)	1% (1%)	0 (0)	0% (0%)	0 (1)	0% (2%)	2 (0)	4% (0%)	3 (2)	2% (1%)
ICS (ceased in parentheses), n, %	0 (1)	0% (1%)	0 (0)	0% (0%)	0 (1)	0% (2%)	1 (2)	2% (4%)	1 (4)	1% (2%)
Any inhaled medication, n, %	16	20%	4	20%	5	12%	6	13%	27	16%
**New inhalers since LHC** (% those with visible record)										
SABA (ceased in parentheses), n, %	26 (0)	33% (0%)	5 (0)	25% (0%)	4 (0)	10% (0%)	6 (0)	13% (0%)	36 (0)	21% (0%)
LAMA/LABA (ceased in parentheses), n, %	28 (1)	35% (1%)	4 (0)	20% (0%)	8 (1)	19% (2%)	6 (0)	13% (0%)	42 (2)	25% (1%)
ICS/LAMA/LABA (ceased in parentheses), n, %	1 (1)	1% (1%)	1 (0)	5% (0%)	0 (0)	0% (0%)	1 (0)	2% (0%)	2 (1)	1% (1%)
ICS/LABA (ceased in parentheses), n, %	2 (0)	3% (0%)	1 (0)	5% (0%)	0 (1)	0% (2%)	1 (0)	2% (0%)	3 (1)	2% (1%)
ICS (ceased in parentheses), n, %	0 (0)	0% (0%)	1 (0)	5% (0%)	0 (0)	0% (0%)	0 (0)	0% (0%)	0 (0)	0% (0%)
Any inhaled medication, n, %	38	48%	9	45%	9	21%	9	20%	56	33%

CRT, community respiratory team; ICS, inhaled corticosteroid; LABA, long acting beta agonist; LAMA, long acting muscarinic antagonist; LHC, Lung Health Check; SABA, short acting beta agonist.

Considering the whole cohort referred to the CRT (n=201), 53 (26%) participants had a record of an inhaler prescription at any time before their LHC. Excluding those participants with subsequent normal spirometry (n=20), 27 individuals were taking some form of inhaled medication at the time of their LHC (16% of all attendees for whom primary care records were accessible), most frequently a SABA. Fifty six participants commenced a new inhaled medication subsequent to the LHC (33% of those with accessible primary care records) with SABA (n=36, 21%) and LAMA/LABA (n=42, 25%) being the most commonly prescribed inhalers. Interestingly, 9 of the 20 participants (45%) where spirometry subsequently excluded COPD remained on inhaled medication at the time of primary care record review (25% SABA, 20% LAMA/LABA). The CAT score of those participants who commenced new inhaled medication subsequent to the LHC was slightly higher than those who did not (mean±SD was 12.9±5.9 vs 10.7±5.7 respectively, p=0.007).

Considering changes in coding and management across the whole population undergoing spirometry as part of their LHC during the study period (n=2391), 201 (8.4%) fulfilled criteria for referral to the CRT of whom 105 (4.4%) were seen (or reviewed earlier by their GP). Excluding those with subsequent normal spirometry, 59 participants had a new COPD code entered into their primary care record (35% of those fulfilling referral criteria, and 2.5% of all those undergoing LHC spirometry) and 56 commenced new inhaled medication (33% of those fulfilling referral criteria and 2.3% of all those undergoing LHC spirometry). Twenty three participants were offered pulmonary rehabilitation by the CRT, of whom five accepted the referral (6.3% of those seen by the CRT with COPD, and 0.2% of all participants undergoing LHC spirometry).

## Discussion

There is clear evidence of underdiagnosis of COPD in the general population,^3 4^ and previous studies have shown a high prevalence of undiagnosed AO in people undergoing LDCT screening.^8 9^ Given the recommendation from GOLD for active case finding in people with symptoms,^7^ the possible roll-out of LCS offers an opportunity to co-deliver spirometry for symptomatic patients with the aim of diagnosing COPD and thus allowing therapeutic interventions earlier in the natural history of the disease. However, a recent USPSTF targeted evidence update found no direct evidence that COPD active case finding improved COPD morbidity, mortality or health-related quality of life.^6^

Other LHC programmes^8 9^ have previously reported a high prevalence of undiagnosed symptomatic AO in people having pre-bronchodilator spirometry alongside LDCT screening. However, the analysis presented here demonstrates that 29% of those having subsequent post-bronchodilator spirometry were not found to have AO, thereby excluding COPD. Most people in our study did not have post-bronchodilator spirometry checked and yet many commenced inhaled treatments for COPD; for some these treatments might be unnecessary. Similarly, 45% of those with confirmed normal post-bronchodilator spirometry continued to receive inhaled medication many months after their assessment. While asthma might be a diagnostic possibility in this group, the majority of patients who remained on inhaled medication were on bronchodilators alone without inhaled corticosteroids, suggesting they may not be on appropriate therapy. Our findings therefore highlight that pre-bronchodilator spirometry alone should not be used to guide treatment, and emphasise the importance of instituting treatment for COPD only after post-bronchodilator spirometry has confirmed this diagnosis.

Of those referred to the CRT, nearly half were not seen either because their referral was not processed or because they declined assessment. For a small number, the referral was affected by the COVID-19 pandemic, but a large number (44) were not invited due to logistical failures in the referral process that pre-dated the pandemic. This highlights the importance of establishing robust referral pathways with regular audit to avoid the failures described here. Nearly a third of people who were invited declined assessment. Previous analysis from YLST has shown clear factors predicting failure to respond to LHC invitation (most notably increased deprivation and current smoking status). However, these factors did not predict refusal to CRT assessment, nor did any other analysed parameter. It may be that these participants have lower symptom burden when compared with people with known COPD and that in the absence of significant functional impairment, a proportion of screening participants are reluctant to engage in further respiratory assessment. This might also explain the low uptake for pulmonary rehabilitation, with only 22% of those offered referral to a pulmonary rehabilitation programme accepting. Although CAT scores did not differ between those who took up the invitation compared with those who did not, there was a significantly higher CAT score in those participants who commenced inhaled medication following their LHC compared with those who did not, although the difference was relatively small.

### Comparison to other published literature

The only previous report of downstream impact of spirometry performed in the context of LCS described outcomes in 55 participants found to have unexplained symptomatic AO from 1542 undergoing screening in a West London pilot.^11^ In that study, participants were advised to see their GP for further assessment and treatment, and 28 (51% of those referred) attended a primary care appointment (four were lost to follow-up and 23 did not attend their GP appointment). Sixteen participants subsequently received a new respiratory diagnosis (14 COPD, 2 asthma, overall 29% of those referred and 1.0% of those screened); pharmacotherapy was commenced in six people (11% of those referred and 0.4% of those screened) and one participant commenced pulmonary rehabilitation (1.8% of those referred and 0.06% of those screened). The corresponding figures reported here (expressed as proportions of those referred and those screened) are: 59 participants with a new GP COPD code (35% and 2.5% respectively); 56 participants commenced appropriate pharmacotherapy (33% and 2.3% respectively) and five participants referred to pulmonary rehabilitation (2.5% and 0.2% respectively).

### Strengths and limitations

A strength of this study is the comparison between pre-bronchodilator LHC spirometry and post-bronchodilator confirmatory spirometry for a proportion of participants (the first study in the context of lung screening to report this). Limitations include the logistical failures in the referral process which resulted in fewer participants benefiting from CRT review; however this service was not part of a clinical trial, and thus represented ‘real-world’ experience. Second, the referral criteria were mostly limited to those participants with an FEV_1_ of less than 80% predicted. This was a pragmatic, although arbitrary criterion to ensure the service was able to cope with demand, and to direct limited resource to those participants who might have the greatest need. However, there may have been people with AO above this threshold who might have benefited from CRT referral, although the spirometry results were communicated to the GP in all cases.

There are ways in which the assessment process and referral criteria might be amended in future programmes. In the current study, only symptomatic patients were referred for further assessment, with self-reporting of any COPD symptom being used to define this group.^13^ An alternative strategy could be to define an appropriate CAT score threshold. This could either be used to determine which patients with AO are referred for further assessment, or possibly could be used to target spirometry testing such that people below this threshold do not undergo this test.

### Summary and implications for practice

This report describes the challenges involved in actioning potential cases of undiagnosed COPD detected by pre-bronchodilator spirometry delivered in the context of LCS. While some of the logistical issues described could easily be addressed (eg, ensuring a robust referral pathway), there are other important points which have implications for future programmes. First, a proportion of participants decline further respiratory assessment (30% here, 42% in West London.^11^ Furthermore some participants who were seen declined onward referral to other services (eg, to pulmonary rehabilitation programmes), perhaps reflecting the low symptom burden experienced by this patient population. Second, 29% of our participants fulfilling referral criteria to the CRT were subsequently found to have no AO following post-bronchodilator spirometry. This most likely reflects the effect of the bronchodilation itself although we cannot exclude a contribution from training/competency issues in measurement of spirometry. The evidence-base for pharmacotherapy is limited to those with post-bronchodilator AO, and thus some participants in the described analysis may be receiving unnecessary treatment (including those with proven normal post-bronchodilator spirometry). Third, many of these participants either self-reported COPD, emphysema or bronchitis (n=43), or had been previously issued with inhaled medication (n=53). In total, 78 (39% of the whole cohort) individuals fulfilled one or other criteria, suggesting possible previous opportunities for diagnosis in primary care.

Adding spirometry to LCS offers the opportunity of earlier diagnosis of COPD and therefore possibly improved outcomes. However, further research is needed to clarify the optimal way to investigate and manage people found to have unexplained symptomatic AO at LHC, and to measure eventual clinical outcomes to confirm overall patient benefit. In addition, there is a need to undertake qualitative research with this population to understand the barriers to patients attending for assessment and treatment of possible COPD, and strategies that might mitigate these to ensure maximum clinical benefit.

## Data Availability

All data relevant to the current manuscript are included in the article. The data sharing policy from the Yorkshire Lung Screening Trial itself are shown below. The Yorkshire Lung Screening Trial is a Clinical Trial (ISRCTN42704678). Data collected as part of this study are available on reasonable request according to the following data sharing policy. 'The YLST study is registered at the ISRCTN registry with identifier ISRCTN42704678. In order to meet our ethical obligation to responsibly share data generated by clinical trials, YLST operates a transparent data-sharing request process. Anonymous data will be available for request once the study has published the final proposed analyses. Researchers wishing to use the data will need to complete a request for data-sharing form describing a methodologically sound proposal. The form will need to include the objectives, what data are requested, timelines for use, intellectual property and publication rights, data release definition in the contract and participant informed consent, etc. A data-sharing agreement from the sponsor may be required'. The data presented here are mostly clinical data subsequent to trial participation, and so is not included in the above data sharing policy.
